# Harnessing exotic germplasm for red clover improvement: Tracking genomic introgression and deploying genomic selection

**DOI:** 10.1002/tpg2.70287

**Published:** 2026-08-18

**Authors:** A. D. Heslop, S. K. Arojju, R. W. Hofmann, J. L. Ford, C. A. Hefer, M. Z. Z. Jahufer, A. C. Larking, W. Hong, T. P. Bilton, R. Ashby, J. O'Connor, A. G. Griffiths

**Affiliations:** ^1^ Faculty of Agriculture and Life Sciences Lincoln University Lincoln New Zealand; ^2^ Bioeconomy Science Institute Lincoln Research Centre Christchurch New Zealand; ^3^ PGG Wrightson Seeds Limited Palmerston North New Zealand; ^4^ Radiata Pine Breeding Company, School of Forestry University of Canterbury Christchurch New Zealand; ^5^ School of Agriculture and Food Sustainability The University of Queensland Brisbane Queensland Australia; ^6^ Bioeconomy Science Institute, Grasslands Research Centre Palmerston North New Zealand; ^7^ Bioeconomy Science Centre, Invermay Agricultural Centre Mosgiel New Zealand

## Abstract

Red clover (*Trifolium pratense* L.) is a globally important temperate forage legume. Its symbiosis with soil‐borne rhizobia enables nitrogen fixation, and its ability to produce quality forage under diverse soil conditions enhances pasture productivity, particularly during water deficits. With increasing climate‐related stresses, harnessing adaptive traits absent in current cultivars is critical. Genebanks conserve diverse red clover germplasm, providing genetic variation for agronomic and adaptive traits. In this study, we introgressed novel germplasm into locally adapted cultivars to track the inheritance of allelic variants using genotyping‐by‐sequencing. Multi‐location, multi‐year trials evaluated half‐sib families (generation two [Gen 2]) two generations removed from the exotic germplasm (genereation zero [Gen 0]) against local cultivars. Several Gen 2 populations matched or outperformed local cultivars and exhibited a moderate family mean heritability (*h* ^2^ > 0.40) for most traits. Integrating genomic, phenotypic, and environmental data, 77 bioclimatic‐associated single nucleotide polymorphisms (SNPs) were identified, of which 35 SNPs and 27 associated genes were significantly linked to trait expression. By using the original germplasm (Gen 0) as a training population and the derived half‐sib families (Gen 2) as a validation population, genomic prediction models were developed to calculate prediction accuracies for key agronomic traits. Biomass and plot density traits showed high predictive abilities and the highest prediction accuracies across generations. This study demonstrates a route by which genetic diversity from genebanks can be successfully incorporated into local populations, enabling evaluation and selection of key traits. The identified molecular markers and genomic prediction models provide a pathway to efficiently develop climate‐adaptive red clover cultivars.

## Core Ideas

- Genotyping‐by‐sequencing tracked valuable alleles from exotic germplasm leading to a better understanding of trait expression and inheritance.
- The discovery of trait‐associated single nucleotide polymorphisms (SNPs) and genes enables future marker‐assisted selection for adaptation.
- The introgression of exotic germplasm led to strong half‐sib agronomic performance.
- The development of genomic prediction models across generations has potential to accelerate red clover improvement.

## Plain Language Summary

Red clover plays an important role in improving pastures through its ability to produce quality biomass under a range of environmental conditions. Through identifying and bringing in adaptive traits from germplasm ecotypes not previously bred from, new cultivars can be produced to maintain this role. In this study, new genetics from ecotypes were bred into local cultivars to track the inheritance of key traits using molecular technology. We combined environmental, genetic, and trait data collected from field trials to evaluate interactions that affect trait expression. This genetic information was then used to predict offspring performance using the germplasm ecotypes as a training population and the second generation of offspring as a validation population within genomic prediction models. This study shows that the diversity from exotic germplasm can provide beneficial traits which, when combined with molecular methodologies and prediction models, can be used to breed new adaptive cultivars.

**Abbreviations**
AAF: alternative allele frequency
BLUPs: best linear unbiased predictors
C‐derived: Colenso derived
GBS: genotyping‐by‐sequencing
GEBVs: genomic estimated breeding values
Gen 0: generation zero
Gen 1: generation one
Gen 2: generation two
GRM: genomic relationship matrix
GWAS: genome‐wide association study
LD: linkage disequilibrium
LSD: least significant difference
MAF: minor allele frequency
MSE: mean squared error
R‐derived: Relish derived
REML: residual maximum likelihood
SNP: single nucleotide polymorphism
SSR: simple sequence repeat

## 1 INTRODUCTION

Red clover (*Trifolium pratense* L.) is an economically important temperate pastoral species worldwide. When incorporated into pastoral systems, red clover can provide forage high in protein and digestibility and grows in a range of soil conditions (Annicchiarico et al., 2015; Taylor & Quesenberry, 1996). As a legume, this species is capable of fixing nitrogen biologically to generate plant‐available nitrogen through symbiosis with soil dwelling *Rhizobia* bacteria species (Taylor & Quesenberry, 1996). Originating from the Eastern Mediterranean region, red clover is now established and utilized throughout most temperate regions worldwide (Annicchiarico et al., 2015) and like many economically important species, collection expeditions have been initiated in regions where red clover is found, with many novel germplasm populations, ecotypes, landraces, and breeding lines now stored worldwide in genebank collections. These germplasm accessions possess a large range of genetic variation for agronomic and adaptive traits. For the development of new adaptive cultivars, harnessing this genetic diversity is important to meet the ever‐changing needs of farmers and to benefit the wider global pastoral farming industry.

The continued development of molecular marker technologies, such as genotyping‐by‐sequencing (GBS), combined with multi‐year multi‐location field evaluation trials has allowed for effective genetic characterization and trait response evaluation of germplasm material. This has provided more robust selection tools when developing core collections or identifying populations that possess beneficial agronomic or adaptive traits (Frey et al., 2022; Heslop et al., 2025; Nay et al., 2023; Zanotto et al., 2021, 2023). Following identification and characterization of germplasm, an important step is to incorporate this material into breeding programs. Introgressing this novel genetic diversity into existing breeding programs, rather than developing a new program, reduces linkage drag by enabling the selection against undesirable traits (Tanksley & McCouch, 1997; Taylor & Quesenberry, 1996). One strategy is to cross the novel material with locally adapted cultivars, producing half‐sib families. By serving as “pollen sinks,” these cultivars enable the tracking, evaluation, and selection of vigor, heritability, and novel traits across following generations.

Rather than relying solely on phenotype information, breeders can in parallel utilize single nucleotide polymorphism (SNP) molecular markers to track desired alleles responsible for adaptation or phenotypic expression. These markers may have been identified through previous genome‐wide association studies (GWASs) (Huang & Han, 2014), landscape genomics (Capblancq & Forester, 2021), or quantitative trait locus mapping (Herrmann et al., 2008) studies. They can also be used to identify, track, and evaluate genetic relationships, trait responses, and mating proficiencies through generations. Using molecular markers alongside phenotypic evaluation allows for simultaneous selection for target traits and agronomic performance, even for complex traits, while minimizing linkage drag (Collard & Mackill, 2008). Previous studies using molecular markers have identified significant SNPs and their associated genes, and evaluated gene expression for establishment, persistence, freezing tolerance, and drought response in red clover (Ergon, 2019; Yates et al., 2014; Zanotto et al., 2023). Similarly, the use of marker‐assisted paternity testing, mainly using simple sequence repeats (SSRs), has been validated and implemented to track inheritance and influence breeding decisions in multiple clover species (George et al., 2018; Riday, 2011; Vleugels et al., 2014, 2019).

One tool that incorporates genome‐wide markers and phenotypic information to enable shorter breeding cycles and increased cost‐effectiveness per unit gain is genomic prediction. By linking associations between molecular markers and phenotype information in a training set of individuals, genomic prediction is used to generate genomic estimated breeding values (GEBVs) for another set of genotyped individuals and is of value particularly for estimating GEBVs within families, where phenotypic information may not be available. As genomic prediction uses genome‐wide markers, it captures most genes responsible for traits of interest, increasing the predictive ability for specific traits (Faville et al., 2012). Currently there are few studies that have validated genomic prediction in red clover under field conditions with the only other published study in red clover evaluating genomic prediction for crude protein, dry matter yield, and date of flowering (Skøt et al., 2024). Higher predictive abilities were found for dry matter yield and date of flowering, along with significant marker × environmental effects that were found to influence predictive ability (Skøt et al., 2024). For white clover (*Trifolium repens* L.), modest predictive abilities were found that could be used to select for dry matter yield, leaf size, growth scores, and stolon density (G. O. Ehoche et al., 2021; O. G. Ehoche et al., 2025). Genomic prediction has also been explored in perennial ryegrass (*Lolium perenne* L.) and alfalfa (*Medicago sativa* L.) both economically important forage species (Annicchiarico et al., 2015; Arojju, Cao, Jahufer, et al., 2020; Blanco‐Pastor et al., 2019; Faville et al., 2018).

Identifying how these traits and genes are inherited and influenced by environmental conditions is crucial for fully utilizing novel germplasm material and ensuring efficient and impactful incorporation into breeding programs. Identification of associations between phenotypic trait response and environmental variables determined through redundancy analysis (Capblancq & Forester, 2021) adds an additional layer for evaluating diverse germplasm material and the development of predictive genomic models for assessing plant performance, trait inheritance, and adaptability to new environments. Recently, the genetic diversity and genetic relationship of a panel of 92 diverse germplasm populations have been characterized where key interactions between plant performance and bioclimatic variables were evaluated (Heslop et al., 2025). In that study several SNPs were identified to be significantly associated with bioclimatic variables that may be used as genetic markers for selection after confirmation of their efficacy. Our objectives were to build upon the previous work to incorporate novel red clover germplasm into breeding lines and use the genetic, phenotypic, and environmental information of this diverse material from low rainfall areas to (i) develop F_2_ half‐sib families from elite pre‐breeding populations derived from top‐crossing exotic red clover germplasm onto locally adapted cultivars; (ii) assess additive genetic variation and heritability for key agronomic and physiological traits among these half‐sib families evaluated in multi‐year multi‐location field trials; (iii) evaluate and validate single‐trait genomic prediction performance models for the assessment of key agronomic and physiological traits across multi‐generational populations evaluated in multi‐year multi‐location field trials; and (iv) identify adaptive genetic markers associated with phenotypic expression of agronomic and physiological traits.

**Core Ideas**

- Genotyping‐by‐sequencing tracked valuable alleles from exotic germplasm leading to a better understanding of trait expression and inheritance.
- The discovery of trait‐associated single nucleotide polymorphisms (SNPs) and genes enables future marker‐assisted selection for adaptation.
- The introgression of exotic germplasm led to strong half‐sib agronomic performance.
- The development of genomic prediction models across generations has potential to accelerate red clover improvement.

## 2 MATERIALS AND METHODS

### 2.1 Plant material, trial design, and trial management

**FIGURE 1:**
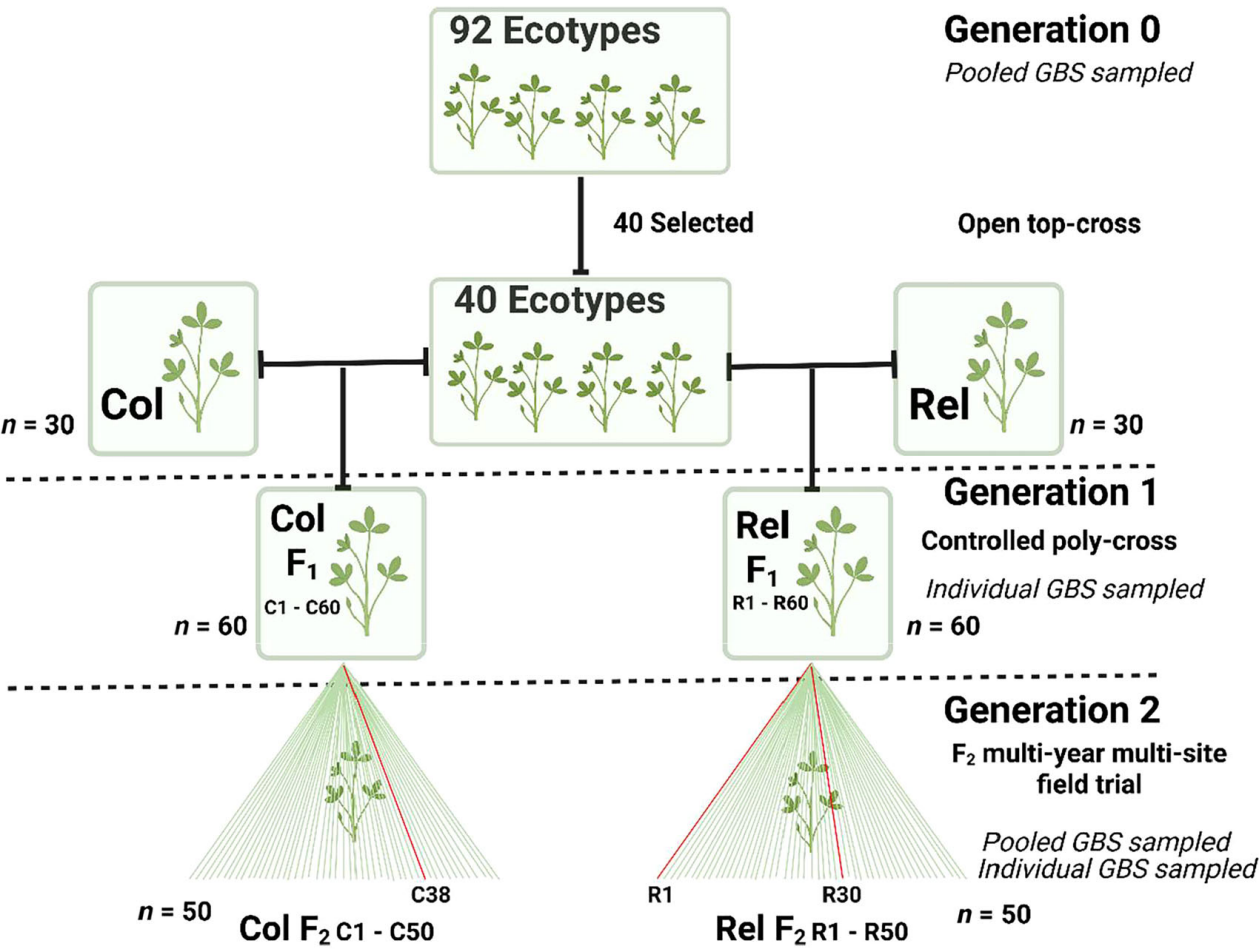
Flow diagram showing the three red clover generations (Generation 0 [Gen 0], Generation 1 [Gen 1], and Generation 2 [Gen 2]) and how they were developed. Note that 40 ecotypes from Gen 0 were top‐crossed to cultivars Colenso (Col) and Relish (Rel) individuals to form 60 cultivar‐derived (Colenso derived [C‐derived] and Relish derived [R‐derived]) Gen 1 families. Of the 60 each C‐derived and R‐derived Gen 1 families, 50 of each were intercrossed in separate poly‐crosses to form 50 Gen 2 half‐sib families. Each of the 40 ecotype populations (Gen 0) underwent genotyping‐by‐sequencing (GBS) using pooled individuals (*n *= 30) for each population. The 60 individuals (Gen 1) in each cultivar‐derived poly‐cross were genotyped individually using GBS, and each of the resultant selected 50 half‐sib families (Gen 2) genotyped by pooled GBS. Additionally, 15 individuals each from three Gen 2 half‐sib families (Rel1 [R1], Rel30 [R30], and Col38 [C38]) were genotyped by GBS. Figure created using Biorender.com.

To collect novel alleles onto locally adapted red clover germplasm, an open topcross was conducted that comprised 40 ecotype populations selected from a larger collection of 92 ecotypes accessed from the Margot Forde Genebank (Bioeconomy Science Institute, Grasslands Research Centre, Palmerston North, New Zealand) whose generation zero (Gen 0; Figure 1) field trial and genomic data are described in Heslop et al. (2025). The 40 ecotypes were topcrossed in the field onto pollen‐recipient locally adapted cultivars. The ecotype populations represented eight countries: Armenia (Arm; 6 populations); Azerbaijan (Azb; 6); Georgia (Geo; 1); Greece (Gre; 6); Portugal (Por; 6); Russia (Rus; 3); Spain (Spa; 6); and Tajikistan (Taj; 6) (Table S1). These ecotypes were selected based on ecogeographical factors, including both place of origin with latitude–longitude coordinates and collection site information along with seed availability. Increased weighting was given to populations collected from low rainfall environments; hence, the selected ecotypes were expected to adapt well under New Zealand's environments. Due to being unexplored germplasm obtained directly from the genebank no information was available on flowering time; therefore, this could not be factored into the selection of the 40 ecotypes. A Gen 0 (Figure 1) field trial of the 40 accessions was established as described in Heslop et al. (2025). Briefly, seed was randomly sampled from each ecotype, then scarified using sandpaper, germinated, and transferred to root trainer pots (3 cm × 3 cm × 10 cm) filled with a peat‐based soil mix. The plants were maintained under glasshouse conditions for 2 months, then transferred outside onto a concrete pad in late winter for “hardening” over 2 months to increase post‐transplanting survival in the field. In autumn 2019, a row‐column field trial was established at the AgResearch Grasslands Research Centre in Palmerston North (40° 21′ S, 175° 37′ E) in Kairanga silt loam (Cowie, 1978). This was established as a two‐replicate monoculture trial with 10 plants of each ecotype hand‐transplanted into 1.5 m rows with 0.25 m spaces between rows.

In the first summer (2019–2020), 30 individuals from two cultivars, Grasslands Colenso (Colenso) and Grasslands Relish (Relish), were grown in 10 L pots. For each cultivar, plants were placed randomly among the field trial during peak flowering when the majority of accessions were flowering to form an open topcross, with pollination occurring naturally from honeybees (*Apis mellifera*), short tongue bumblebees (*Bombus subterraneus*), and long tongue bumblebees (*Bombus hortorum*). After a period of 10 days each in the field, the cultivar representatives were moved into bee‐proof isolation cages to allow seed development. Colenso was the first cultivar in the topcross, followed by Relish. At maturity, ripe seeds from each of the 30 plants per cultivar were harvested and maintained separately. Equal amounts of seed from 15 individuals with the highest number of seed for each cultivar were combined together to form a balanced bulk. This resulted in the generation of two F_1_ balanced bulks, Colenso‐derived (C‐derived) F_1_ and Relish‐derived (R‐derived) F_1_ from Colenso and Relish cultivars, respectively. This is generation one (Gen 1) (Figure 1).

In spring 2021, a random sample of 60 seeds from each F_1_ balanced bulk (Gen 1) was scarified and germinated, as described above, and transferred to PB3 pots (10 cm × 10 cm × 20 cm) filled with a peat‐based soil mix. The 120 potted plants, 60 representing each of the two F_1_ bulks, were maintained under glasshouse conditions for a period of 2 months. Prior to flowering, 60 individuals representing each F_1_ breeding line were transferred into two separate bee proof crossing cages for poly‐crossing to generate F_2_ breeding lines (generation two [Gen 2]; Figure 1). For pollination, pollen‐free long tongue bumblebees (*Bombus hortorum*) were introduced to each crossing cage as flowering required. Once harvested separately, the 50 top seed‐producing F_2_ individuals from each breeding line were selected to take to field trials. The seed harvested from these 50 F_2_ individuals formed the two half‐sib F_2_ family‐based populations (Gen 2).

As described above, a random sample of seed from each of the 50 F_2_ individuals for each cultivar‐derived family was scarified, germinated, and transferred to root trainer pots, grown under glasshouse conditions for 2 months, then “hardened” outside on a concrete pad in late winter over 2 months. In spring 2022, row‐column design‐based field trials with two replicates were established at two locations (described below), Lincoln, Canterbury, New Zealand (43° 38′ S, 172° 30′ E) and Brookside, New Zealand (43° 70′ S, 172° 32′ E). Fifteen plants of each F_2_ half‐sib population were hand‐transplanted into each plot. Plots were 1 m × 1 m with inter‐plot spacings of 1 m. Both trials were established in September (Spring) 2022 and continued until March (Autumn) 2025.

The Lincoln site was situated on Wakanui silt loam soil (Brown et al., 2005), 14 m above sea level. The annual rainfall for Years 1, 2, 3 and for the combined years was 803, 571, and 383 mm totaling 1757 mm. The Brookside site was situated close to a creek bed on Templeton silt loam soil (Martin, 1990), 29 m above sea level. The annual rainfall for Years 1, 2, 3 was 719, 541, and 277 mm, respectively, and totaling 1537 mm. Due to the close proximity of the two sites, temperature information was shared between sites with mean maximum temperature for Years 1, 2, 3 and overall were 18.7°C, 19.5°C, 18.8°C, and 19°C, respectively. Mean minimum temperature for Years 1, 2, 3 and overall were 8.2°C, 7.1°C, 8.9°C and 8.1°C, respectively. Both locations were sown with a diploid perennial ryegrass (*Lolium perenne* L.) cultivar Ceres One50 containing the *Epichloë festucae* fungal endophyte AR37 strain to mimic a mixed sward cattle/sheep grazed management.

At both locations, standard farming practices were followed by rotationally grazing the trials when herbage mass was between 2500 and 2800 kilograms of dry matter per hectare (kg DM ha^−1^), as assessed by a rising plate meter. Animals were removed immediately when pasture residuals were between 1000 and 1200 kg DM ha^−1^. Each grazing was for a period of 1–2 days, season dependent, to ensure rapid and uniform defoliation and limit the effect of urine patches in the trials. Trials were rotationally grazed by sheep at Lincoln and cattle at Brookside. Both sites were run as dryland using only natural precipitation after the first year. For a period of 3 months immediately after transplanting, irrigation was applied to help with establishment at the Brookside site.

### 2.2 Morphological and physiological traits

Five morphological and physiological traits were assessed at least seasonally each year at both Lincoln and Brookside sites. Qualitative visual scores were used to measure (i) biomass and (ii) plant plot density based on a 1 (very low) to 9 (very high) scale, with 1‐unit increments, where a score of 5 represented an average plot relative to the trial. Biomass scores reflected relative aboveground biomass accumulation, with low scores indicating minimal biomass and higher scores indicating greater biomass. Plot density scores were referenced to approximate ground cover (e.g., 1 = 10% coverage, 9 = 90% coverage). (iii) Average leaf size per plot was measured using a qualitative visual 1 (low) to 5 (high) scale, with 1unit increments. (iv) Relative chlorophyll content was measured using a Konica Minolta SPAD‐502Plus chlorophyll meter. An average reading was taken from three randomly chosen young, fully unfolded leaves per plant (Ling et al., 2011). (v) Plant survival was a visual count of survivors within each plot of 15 plants; this was completed once per year. All traits were measured immediately prior to grazing.

### 2.3 Additive variance and narrow sense heritability

A linear mixed model was fitted for the five traits using DeltaGen analysis software (Jahufer & Luo, 2018), which employs a residual maximum likelihood (REML)‐based approach using the “*lme4*” R package (Bates et al., 2015). This analysis generated estimates of additive genetic variance components, variance among the half‐sib families, and estimates for best linear unbiased predictors (BLUPs) for each trait. The following linear model was fitted. The model was used as a completely random model as well as a mixed model, depending on the analysis conducted:

$$\begin{array}{ccc} Y_{inojklm} & = & \mu +p_{i}+y_{o}+e_{n}+{\left(pe\right)}_{in}+s_{j}+{\left(ps\right)}_{ij}+{\left(py\right)}_{io}\\ & & +\;{\left(sy\right)}_{jo}+{\left(pey\right)}_{ino}+{\left(psy\right)}_{ioj}+b_{nojk}+r_{nojkl}\\ & & +\;c_{nojkm}+e_{inojklm} \end{array}$$

where *Y_inojklm_* is the value of a trait measured from population *i*, at the *n*th location of the *o*th year in the *j*th season, within the *k*th replicate, in *l*th row, and *m*th column. *i* = 1,…, *n_p_*; *n* = 1,…, *n_e_*; *o* = 1,…, *n_y_*; *j* = 1,…, *n_s_*; *k* = 1,…, *n_b_*; *l* = 1,…, *n*_r_; *m* = 1,…, *n_c_*; where *p*, *y*, *e*, *s*, *b*, *r*, and *c* are populations, years, locations, seasons, replicates, rows, and columns, respectively. *μ* is the overall mean; *p*_i_ is the random effect of population *i* and distributed as *N*(0, *σ* ^2^ *_p_*); *y_o_* is the fixed effect of year *o*; *e_n_* is the fixed effect of location *n*; (*pe*)*_in_* is the random effect of the interaction between population *i* and location *n*, 𝑁(0, 𝐼σ^2^_g𝑒_); where *I* is the identity matrix, *s_j_* is the fixed effect of season *j*, *N*(0, 𝐼σ^2^_𝑠_); (*ps*)_ij_ is the random effect of the interaction between population *i* and season *j*, *N*(0, 𝐼σ^2^_g𝑠_); (*py*)*_io_* is the random effect of the interaction between population *i* and year *o*, *N*(0, 𝐼σ^2^_𝑖𝑜_); (*sy*)*_jo_* is the random effect of the interaction between season *j* in year *o*, *N*(0, 𝐼σ^2^ *_𝑠𝑦_*); (*pey*)*_ino_* is the random effect of the interaction between population *i* location *n* and year *o*, *N*(0, 𝐼σ^2^ *_gey_*); (*psy*)*_ioj_* is the random effect of the interaction between population *i* season *j* and year *o*, *N*(0, 𝐼σ^2^ *_𝑖𝑜𝑗_*); *b_nojk_* is the random effect of replicate *k* within season *j* within year *o* in location *n*, 𝑁(0, 𝐼σ^2^ *_𝑏_*); *r_nojkl_* is the random effect of row *l* within replicate *k* in season *j* within year *o* in location *n*, *N*(0, 𝐼σ^2^_𝑟_); *c_nojkm_* is the random effect of column *m* in replicate *k* within season *j* within year *o* in location *n*, *N*(0, 𝐼σ^2^_𝑐_); and *e_inojklm_* is the residual effect of population *i* in location *n* within year *o* in season *j* of replicate *k* in row *l* and column *m* of replicate *k* in season *j* within year *o* in location *n*. This produced REML estimates of the variance components for each trait and genomic best linear unbiased prediction values for each population.

The variance components estimated using the linear mixed model analysis were used to calculate a line mean narrow sense heritability for each trait using different adaptations of the following equation:

$$\begin{array}{ccc} & & \;h_{n}^{2}=\\ & & \frac{\sigma_{p}^{2}}{\left(\sigma_{a}^{2}\right)+\left(\sigma_{\mathrm{ab}}^{2}\right)/n_{r}+\left(\sigma_{\mathrm{as}}^{2}\right)/n_{s}+\left(\sigma_{\mathrm{ay}}^{2}\right)/n_{y}+\left(\sigma_{\mathrm{al}}^{2}\right)/n_{l}+\left(\sigma_{e}^{2}\right)/\left(2\times 4\times 3\times 2\right)} \end{array}$$

where *h^2^_n_* is line mean narrow sense heritability. In the estimation of narrow sense heritability; *σ* ^2^ *_a_* was the estimated additive genetic variation among populations; *σ* ^2^_ab_ is the variance associated with the population‐by‐replicate interaction; *n*_r_ was the number of replicates; *σ* ^2^_as_ is the variance associated with population‐by‐season interaction; *n*_s_ was the number of seasons; *σ* ^2^_ay_ is the variance associated with population‐by‐year; *n*_y_ was the number of years; *σ* ^2^_al_ is the variance associated with adaptive genetic‐by‐location interaction; *n*_l_ was the number of years; and *σ* ^2^_e_ is the variance of residuals (Arojju, Cao, Trolove et al., 2020).

### 2.4 DNA isolation and genotyping of bulked samples

Genotype and allele frequency data for the 92 populations (Gen 0) were generated in a previous publication (Heslop et al., 2025) and were derived from pools of 30 individual plants per population. The same procedure was applied for F_1_ individuals (Gen 1) and their corresponding F_2_ half‐sib families (Gen 2) (Figure 1), with the only difference being individual samples were taken for F_1_ material. Pooled samples were taken for all F_2_ half‐sib families along with some individual samples (*n* = 15) for each of one Colenso (Col38) and two Relish (Rel1 and Rel30) derived F_2_ families, respectively (Figure 1). This mixed GBS strategy was adopted to efficiently capture population‑level allele frequencies in a highly heterozygous, outcrossing species, where substantial within‑population genetic diversity is expected, while remaining within practical cost constraints. This is because GBS of 30 individuals to represent allelic diversity per population rather than a pooled sample of 30 individuals with four technical replicates would represent ∼7.5‐fold greater genotyping costs. Individual genotyping of the Gen 1 parents (*n* = 60 each for Colenso [Col] and Relish [Rel]) was undertaken to facilitate controlled crossing and to enable assessment of parental contributions, providing a validation point for inferences drawn from pooled allele frequency data.

In short, DNA was extracted from seedlings as described (Anderson et al., 2018) and GBS libraries were generated in 96‐plex using a protocol adapted from Griffiths et al. (2019) with modifications such as digestion with *Pst* I restriction enzyme and inclusion of four technical replicates for each population as described (Faville et al., 2020). GBS libraries were single‐end sequenced (100 bp reads) using two lanes (∼44 Gb data) on an Illumina HiSeq 2500 (Illumina) at AgResearch Invermay. Sequence reads were quality‐checked, demultiplexed and mapped to the ARS RC 1.1 (GCA_020283565.1) red clover reference genome (Bickhart et al., 2022). SNPs were detected and filtered accordingly (Heslop et al., 2025).

Allele frequencies were combined across technical replicates and were an estimate of the allele frequency at a SNP for the pooled samples or kept separate for individuals. Allele frequencies were extracted and a frequency of one indicated homozygosity of the population for the reference allele, an allele frequency of zero indicated homozygosity of the alternative allele. For analysis, missing data of the filtered SNPs with >95% coverage were replaced by mean allele frequency across populations at the given SNP position (Heslop et al., 2025).

The alternative allele frequency (AAF) was calculated by using the following equation:

$$\mathrm{AAF}=\mathrm{Alt}/\left(\mathrm{Alt}+\mathrm{Ref}\right)$$

where Alt is the alternative allele count, and Ref is the reference allele count (Li, 2011).

### 2.5 Genomic relationship matrix

A genomic relationship matrix (GRM) was created from information sourced from the AAF by using the r package “GUSrelate” (genotyping uncertainty with sequencing data for relatedness) (Bilton et al., 2024). The GUSrelate package filters SNPs based on quality, accounting for minor allele frequencies through a designated threshold and missing data to construct a GRM. A minor allele frequency (MAF) threshold of 0.01 was used for this study. Discriminant analysis of principal components analysis performed using the r package “adegenet” presented by Heslop et al. (2025) of Gen 0 individuals was used to identify the ancestry proportions of the Gen 1 individuals (Jombart, 2008; Jombart et al., 2010, 2018). This was undertaken through the alignment of SNPs from both the reference population (Gen 0) and offspring (Gen 1) AAF matrices and used nonnegative least squares to estimate the ancestry proportions of each offspring based on the reference panel. To account for the diploid nature of red clover, alleles were combined by multiplying AAFs by two to fit a “0/0, 0/1, 1/1” structure and then recoded to a −1 to 1 scale. The genotype similarity was used to predict which male from Gen 1 most likely fathered each individual from the three Gen 2 families (Relish 1, Relish 30, and Colenso 38) using the R package “dplyr” (Wickham et al., 2025).

### 2.6 Ancestry analysis and linkage disequilibrium

From previous analyses (Heslop et al., 2025), 122 SNPs and their associated genes, identified in relation to bioclimatic variables, underwent further exploration. For each SNP, a SNP × trait association analysis was performed to highlight significant (*p* < 0.01) associations between detected SNPs and phenotypic trait expression information gathered from field trials in the Gen 2 pooled half‐sib families using the “vcfR,” “dplyr,” and “rrBLUP” R packages (Endelman, 2011; Knaus et al., 2023; Wickham et al., 2025). For each trait the five top, middle, and bottom performing families for C‐derived and R‐derived populations were combined to make three groups, a top combined (C‐derived top and R‐derived top), middle combined, and bottom combined group. For each group and trait combination a linear regression was run, which generated *p*‐values and effect sizes. SNPs that showed a significant (*p* < 0.01) association with phenotypic expression were filtered, and the effect size and direction were extracted for alternate allele influence. Mapping of the SNPs, associated genes, and phenotypic traits was completed in PhenoGram (Wolfe et al., 2013).

Genotype probabilities were obtained from SNP data processed with the “updog” package (Gerard et al., 2018). SNPs were filtered to retain those with a MAF ≥ 0.05, missing data ≤ 10%, and a minimum read depth of 30 per SNP per individual. Genotype probabilities were converted to expected allele dosages (0–2) per individual. These dosages were then converted to a binary genotype matrix using the “snpStats” package (Clayton, 2012) in R. Pairwise linkage disequilibrium (LD) among SNPs was estimated as the squared correlation coefficient (*r* ^2^) using the ld () function. LD decay was assessed by calculating the mean *r* ^2^ between SNP pairs as a function of their physical distance (in base pairs), with decay distance defined as the first point where the mean *r* ^2^ dropped below 0.2.

### 2.7 Genomic prediction

Genomic prediction was performed using the R package “rrBLUP” (Endelman, 2011) to generate GEBVs for multiple traits. This used the GRM and phenotypic BLUPs for Gen 0 ecotype populations, which were used as our training set. This allowed the evaluation of the relationship between phenotype and genotype of the pooled genotypes, while also allowing the prediction of trait performance of offspring based solely on their genotype and genetic similarity to the training set. To evaluate the model performance across traits, a cross‐validation (2000 iterations per trait) was completed where 20% of genotypes are treated as missing and their breeding values are predicted using the remaining genotypes with phenotype information. For each trait, prediction accuracy (Pearson correlation between predicted and observed values), dispersion bias (slope of observed vs. predicted), and mean squared error (MSE) were calculated to help identify predictive power for each trait. After validation, the prediction model (2000 iterations per trait) based on Gen 0 pooled genotypes was used to produce GEBVs to assess phenotypic performance of all Gen 2 pooled half‐sib families. These predicted GEBVs were compared to actual phenotypic data of all Gen 2 pooled half‐sib families to assess the predictive accuracy of the model.

## 3 RESULTS

### 3.1 Plant performance and trait heritability across generations

**FIGURE 2:**
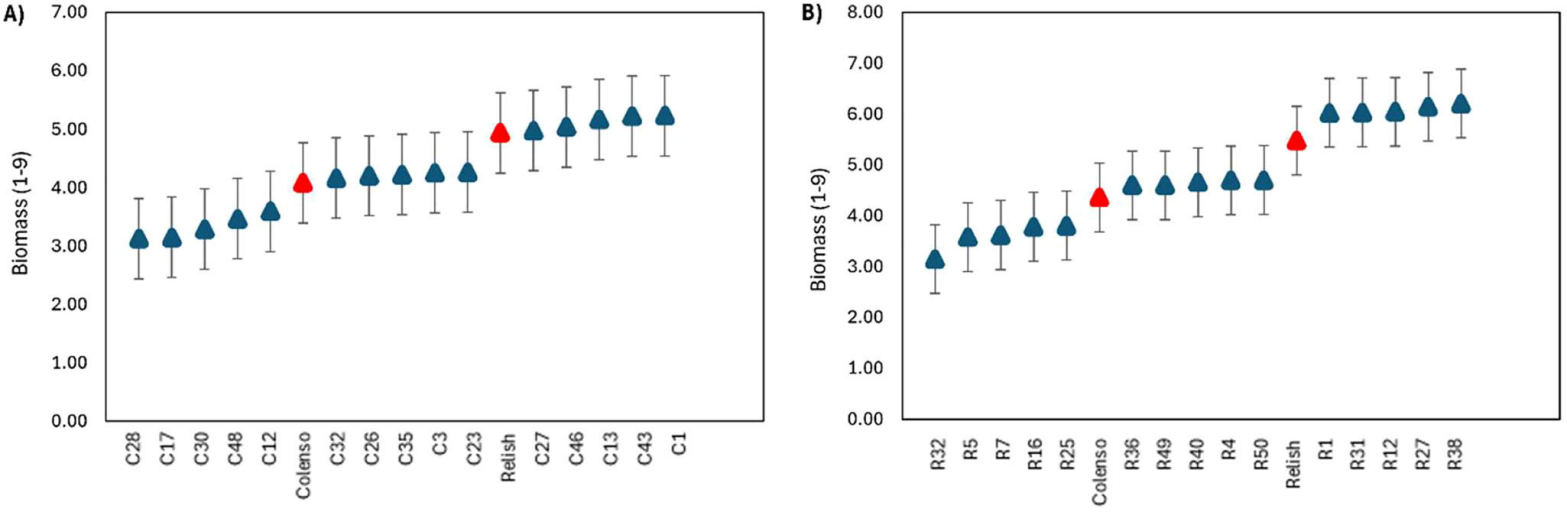
Biomass score BLUPs across locations, years, and seasons for top, middle, and bottom five performing generation two (Gen 2) half‐sib families derived from exotic red clover germplasm crossed onto locally adapted cultivars. (A) Colenso‐derived populations relative to red clover cultivars Colenso and Relish. (B) Relish‐derived populations relative to Colenso and Relish. Error bars represent ± least significant differences (LSDs).

Agronomic performance in the form of growth biomass was evaluated for each C‐derived and R‐derived population alongside Colenso and Relish cultivar checks. The top, middle, and bottom‐performing five populations for biomass scores across locations, years, and seasons are presented in Figure 2. Agronomic data for biomass for all populations are presented in Figure S1A,B. For the C‐derived populations, most performed similarly to the parent Colenso cultivar, including seven populations that exhibited higher mean biomass scores. These differences, however, did not exceed the average least significant difference (LSD) value, indicating limited statistical separation (Figure 2A and Figure S1A). For the R‐derived material, 40 of the 50 populations performed similarly to the parent Relish cultivar with eight having significantly lower biomass production (Figure 2B and Figure S1B). Seven R‐derived populations exhibited higher mean biomass scores than Relish; however, these differences were not consistently greater than the average LSD value, indicating limited statistical separation (Figure S1B). These seven did, however, have significantly greater biomass than Colenso. None of the R‐derived populations had significantly lower biomass than Colenso.

Estimated additive genetic variance (*σ* ^2^ *_a_*) and narrow sense heritability (*h* ^2^) were calculated individually for both C‐derived and R‐derived Gen 2 half‐sib families. Significant (*p* = 0.05) additive genetic variance components and associated narrow sense heritability estimates are presented in Table 1. Among populations similar heritability trends were found for most traits. Both yearly biomass and plot density scores had the highest heritability trends (0.45–0.81) with chlorophyll content and leaf size scores being lower (0.2–0.67). Across all measurements, the seasonal traits had lower heritability values. Additive variance and narrow sense heritability values for Gen 0 material were obtained from Heslop et al. (2025) to enable intergenerational comparisons. Across generations, narrow sense heritability estimates showed similar trends across traits with biomass and plot density traits again having the higher estimates.

**TABLE 1:** Significant estimated additive genetic variance (*σ* ^2^ *_a_*), narrow sense heritability (*h* ^2^) and associated standard error (±) for Colenso‐derived [C‐derived] and Relish‐derived [R‐derived] generation two (Gen 2) populations and generation zero (Gen 0) for five morphological and physiological traits across years and seasons. Heritability values for Gen 0 were from Heslop et al. (2025).

	C‐derived Gen 2	R‐derived Gen 2	Gen 0	C‐derived Gen 2	R‐derived Gen 2	Gen 0	C‐derived Gen 2	R‐derived Gen 2	Gen 0	C‐derived Gen 2	R‐derived Gen 2	Gen 0	C‐derived Gen 2	R‐derived Gen 2	Gen 0
	Biomass			Plot density			Chlorophyll content			Leaf size			Survival		
**All**															
*σ* ^2^ *_a_*	0.19 ± 0.43	0.34 ± 0.09	0.86 ± 0.20	0.23 ± 0.06	0.44 ± 0.11	1.14 ± 0.21	0.64 ± 0.26	0.10 ± 0.34	3.67 ± 0.78	0.02 ± 0.01	0.01 ± 0.01	0.38 ± 0.07	0.76 ± 0.26	1.20 ± 0.41	4.61 ± 0.90
*h* ^2^	0.69	0.74	0.7	0.81	0.81	0.84	0.56	0.67	0.7	0.43	0.43	0.85	0.63	0.68	0.73
**Year 1**															
*σ* ^2^ *_a_*	0.31 ± 0.09	0.25 ± 0.08	1.44 ± 0.27	0.20 ± 0.08	0.23 ± 0.08	1.09 ± 0.23	ns	ns	ns	ns	ns	ns	ns	ns	ns
*h* ^2^	0.69	0.64	0.82	0.52	0.64	0.71	ns	ns	ns	ns	ns	ns	ns	ns	ns
**Year 2**															
*σ* ^2^ *_a_*	0.30 ± 0.09	0.72 ± 0.19	1.08 ± 0.25	0.28 ± 0.09	0.71 ± 0.18	1.68 ± 0.32	0.52 ± 0.32	1.32 ± 0.44	3.34 ± 0.90	ns	ns	ns	ns	ns	ns
*h* ^2^	0.47	0.64	0.66	0.45	0.66	0.82	0.2	0.42	0.49	ns	ns	ns	ns	ns	ns
**Year 3**															
*σ* ^2^ *_a_*	0.24 ± 0.10	0.42 ± 0.15	1.10 ± 0.28	0.23 ± 0.11	0.35 ± 0.19	1.58 ± 0.29	ns	ns	ns	ns	ns	ns	ns	ns	ns
*h* ^2^	0.49	0.57	0.63	0.46	0.27	0.82	ns	ns	ns	ns	ns	ns	ns	ns	ns
**Autumn**															
*σ* ^2^ *_a_*	0.20 ± 0.11	0.44 ± 0.15	1.14 ± 0.24	0.24 ± 0.14	0.50 ± 0.21	1.01 ± 0.23	ns	ns	ns	ns	ns	ns	*∼*	*∼*	*∼*
*h* ^2^	0.35	0.63	0.72	0.41	0.64	0.67	ns	ns	ns	ns	ns	ns	*∼*	*∼*	*∼*
Spring															
*σ* ^2^ *_a_*	0.16 ± 0.07	0.59 ± 0.16	1.01 ± 0.23	0.17 ± 0.07	0.55 ± 0.15	1.18 ± 0.24	ns	ns	ns	ns	ns	ns	*∼*	*∼*	*∼*
*h* ^2^	0.39	0.69	0.66	0.37	0.68	0.71	ns	ns	ns	ns	ns	ns	*∼*	*∼*	*∼*
**Summer**															
*σ* ^2^ *_a_*	0.16 ± 0.05	0.37 ± 0.09	0.87 ± 0.26	0.31 ± 0.10	0.38 ± 0.13	1.17 ± 0.29	0.70 ± 0.42	0.70 ± 0.44	4.82 ± 1.20	ns	ns	ns	*∼*	*∼*	*∼*
*h* ^2^	0.5	0.5	0.57	0.09	0.5	0.69	0.2	0.2	0.65	ns	ns	ns	*∼*	*∼*	*∼*

*Note*: Nonsignificant relationships = ns, trait not measured = ∼.

### 3.2 Ancestry analysis

As previously reported by Heslop et al. (2025), the 40 ecotypes (Gen 0) used in the development of the populations to generate half‐sib families in this current study had been assigned to seven eco‐geographical clusters. In short, the geographic placement of clusters was Cluster 1 (Northern Spain/Portugal), Cluster 2 (a cosmopolitan group from the Caucasus region/Central Europe to the United Kingdom), Cluster 3 (Armenia, Azerbaijan, and Caucasian Russia), Cluster 4 (Tajikistan), Cluster 5 (Greece), Cluster 6 (Armenia, Georgia, Caucasian Russia, and Eastern Turkey), and Cluster 7 (Northern Spain, Portugal, Morocco, and Greece). The 40 ecotypes used in this breeding material were spread across these clusters with six ecotypes from Cluster 1, five from Cluster 2, 11 from Cluster 3, six from Cluster 4, six from Cluster 5, two from Cluster 6, and four from Cluster 7. Of the 4508 SNPs identified previously in Gen 0 individuals (Heslop et al., 2025), 3275 were present in Gen 1 and Gen 2 individuals, and these SNPs underpinned the following genetic analysis.

**FIGURE 3:**
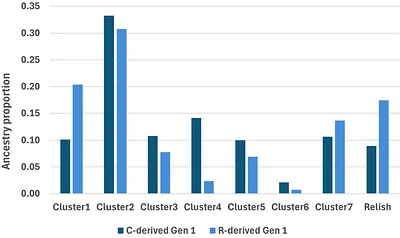
The average proportion inherited from a field trial‐based topcross of generation zero (Gen 0) exotic ecotypes and Relish (control cultivar) found in red clover Colenso‐derived (C‐derived) generation one (Gen 1) (*n* = 60) and Relish‐derived (R‐derived) Gen 1 (*n* = 60) individual parents of the generation two (Gen 2) half‐sib families, grouped by seven clusters identified through discriminant analysis of principal components (DAPC) analysis from Heslop et al. (2025).

From the open topcrosses in the field, the average proportion of SNPs inherited from the Gen 0 ecotypes found in the C‐derived Gen 1 individuals (*n* = 60) that were parents of the Gen 2 half‐sib families was 10%, 33%, 11%, 14%, 10%, 2%, and 11% from Clusters 1 to 7, respectively (Figure 3). The average proportion from Relish, the only cultivar present as an entry in the field trial where the open topcross occurred, was 9%. Four ecotype populations, Azb13 and Por49 (Cluster 2), Taj77 (Cluster 4), and Por52 (Cluster 7), were found to contribute the highest proportion to C‐derived Gen 1 individuals (Figure 3). The average proportion inherited from the Gen 0 ecotypes found in the R‐derived Gen 1 individuals (*n* = 60) was 20%, 31%, 8%, 2%, 7%, 1%, and 14% from Clusters 1–7, respectively. Except for the larger proportion inherited from Cluster 1, these values were similar to those in the C‐derived individuals. As expected in these R‐derived individuals, the average genomic proportion attributed to Relish was 14%, higher than the 9% seen in C‐derived Gen 1 individuals (Figure 3). Five ecotype populations, Por53 (Cluster 1) and Azb13, Rus20, Por49, and Spa64 (Cluster 2), were found to contribute the highest proportion to R‐derived Gen 1 individuals (Figure 3). Average proportion inheritance for all Gen 1 individuals is available (Tables S2 and S3).

When looking at the top five and bottom five Gen 1 individuals for agronomically important traits, biomass, plot density, and survival scores across years and seasons inferred by the performance of their corresponding half‐sib offspring (Gen 2 individuals), the proportion inherited from Gen 0 ecotypes found in C‐derived Gen 1 and R‐derived Gen 1 individuals did not provide insight into what could be causing improved or below‐average performance for each trait. This may be due to the differences of the genetic make‐up of top and bottom five individuals for each trait not being significantly different to the average genetic make‐up of the total 50 families (Figure S2). Interestingly, Cluster 2 showed the highest ancestry contribution to both C‐derived and R‐derived populations. This pattern may have been influenced by flowering synchrony during the original open topcross. The shared genetic background between these populations may therefore have reduced the ability to distinguish trait differences attributable to genetic effects.

GBS genotype data were generated for 29, 30, and 26 individual half‐sib family members from each of three Gen 2 half‐sib families collected from Gen 1 mother plants Colenso 38 (C38), Relish 1 (R1), and Relish 30 (R30), respectively (Figure 1), and mating proficiency was calculated. This was to estimate how many Gen 1 parents were represented as pollen donors for Gen 2 half‐sib families (Figure 1 and Figure S3). For Gen 2 half‐sib family C38, of the 49 other Gen 1 Colenso contributors in the poly‐cross, 11 individuals contributed to at least one family member, and two Gen 1 parents were estimated to have contributed to two or more C38 half‐sib family members. Gen 1 C36 and C46 were the most prolific pollen donors to C38 half‐sib progeny with eight and six estimated, respectively. For Gen 2 half‐sib family R1, of the 49 other Gen 1 Relish contributors in the poly‐cross, 19 individuals contributed to at least one R1 half‐sib family member. For Gen 2 half‐sib family R30, five Gen 1 parents contributed to at least one R30 half‐sib family member. Gen 1 R31 and R14 parents contributed to four and five Gen 2 R30 family members, respectively, while R37 was a prolific pollen donor to 11 of the 26 individual Gen 2 R30 family members.

### 3.3 LD and significant SNP associations revealed

**FIGURE 4:**
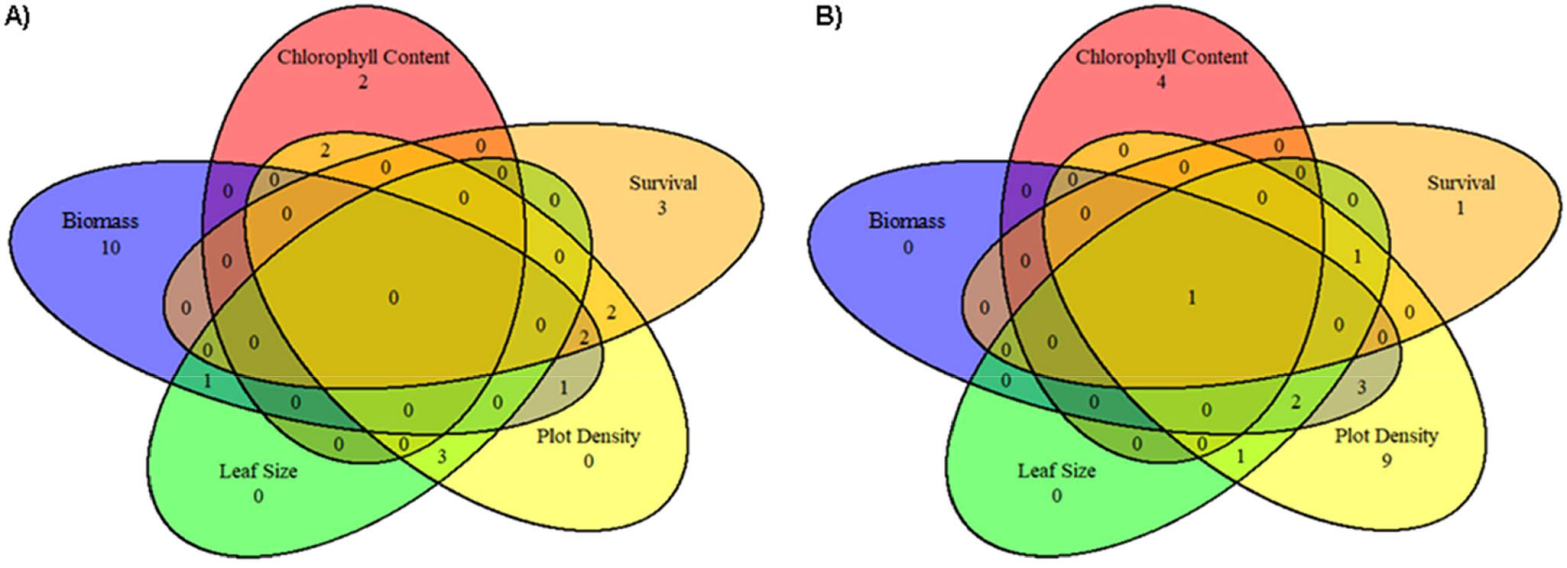
(A) The number of significant bioclimatic single nucleotide polymorphisms (SNPs) identified to have associations with the expression of growth biomass, plot density, leaf size, chlorophyll content, and survival traits for top‐performing half‐sib families. (B) The number of significant bioclimatic SNPs identified to have associations with the expression of biomass, plot density, leaf size, chlorophyll content, and survival traits for bottom‐performing half‐sib families.

**FIGURE 5:**
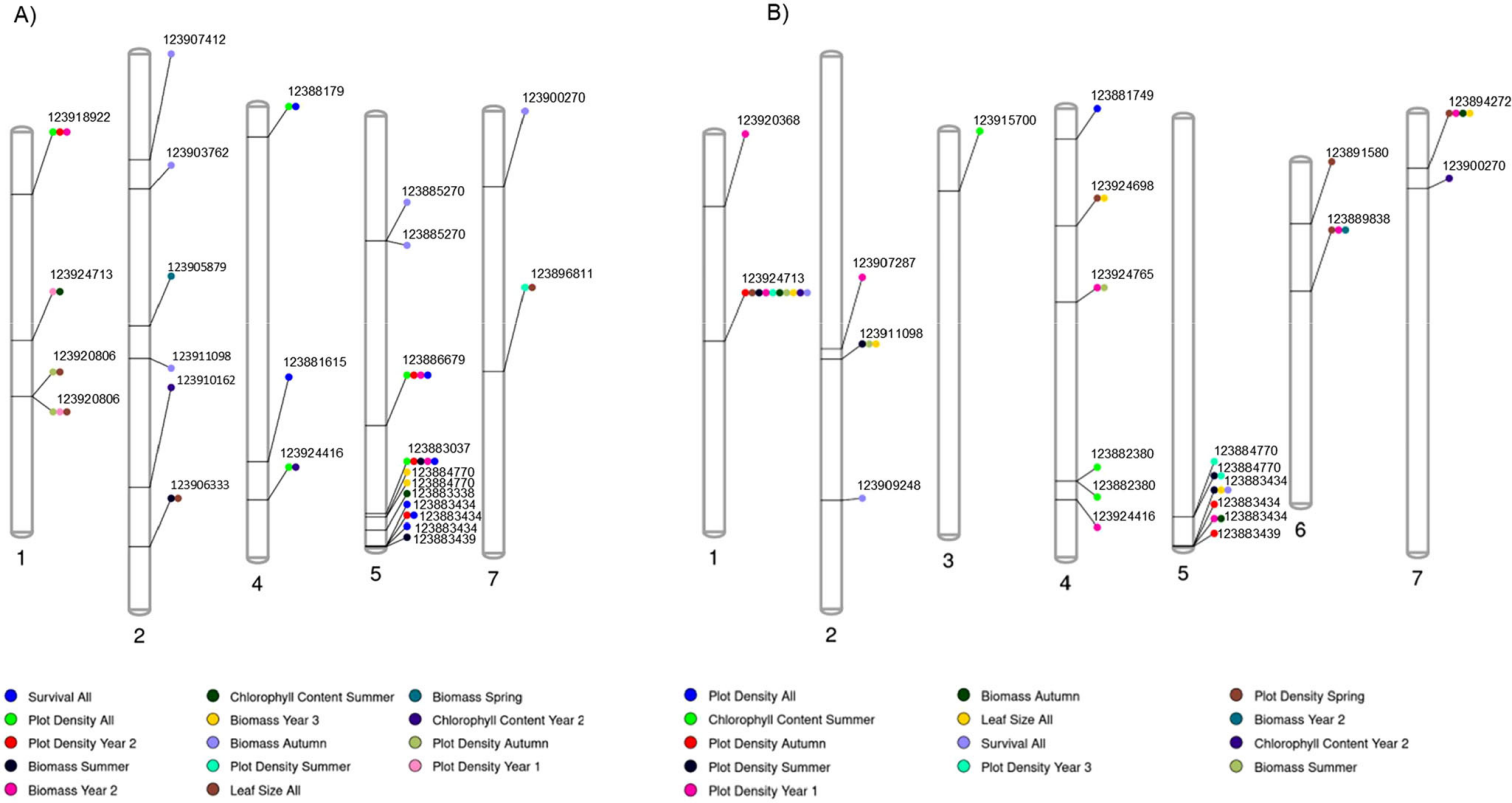
The location of identified trait‐associated single nucleotide polymorphisms (SNPs) on the red clover consensus chromosome map assembled by Bickhart et al. (2022). The phenotypic trait shown to have significant association with the SNPs is indicated by colors. (A) top five individuals and (B) bottom five individuals. The numbers refer to the gene identifier based on annotation from Bickhart et al. (2022). Some loci and genes contain multiple SNPs and trait associations as identified by trait colors and locus positions.

Of the 123 SNPs previously identified in Gen 0 as being significantly associated with key bioclimatic variables (Isothermality, Bio3; minimum temperature of the coldest month, Bio6; and mean temperature of driest quarter, Bio8) (Heslop et al., 2025), 77 were present in the Gen 1 individuals and Gen 2 half‐sib populations. Analysis to identify significant SNP × trait associations using these 77 SNPs was performed for all five traits across years and seasons observations (*n* = 19; Table 1) in the top five and bottom five performing C‐derived and R‐derived Gen 2 half‐sib families. A total of 35 SNPs had a significant (*p* < 0.01) trait association, and of these, 26 and 22 were in the top‐ and bottom‐performing Gen 2 half‐sib families, respectively (Table S4). Distribution of these SNPs among the trait observations was relatively similar among the top‐ and bottom‐performing families with some SNPs shared among traits. A major difference was that in the top families, nearly half the 26 SNPs (10) were associated with biomass only, and none were associated with plot density only (Figure 4A). This was reversed in the bottom families where of the 22 SNPs, most (nine) were associated solely with plot density and none solely with biomass traits (Figure 4B). The location of these 26 and 22 SNPs among the chromosomes of the top‐ and bottom‐performing families, respectively, is shown in Figure 5. These SNPs were spread across the genome with some commonality of SNPs among the top‐ and bottom‐performing families. For both sets of families, there was a cluster of SNPs associated with many of the biomass traits at the bottom of chromosome 5 (Figure 5).

**FIGURE 6:**
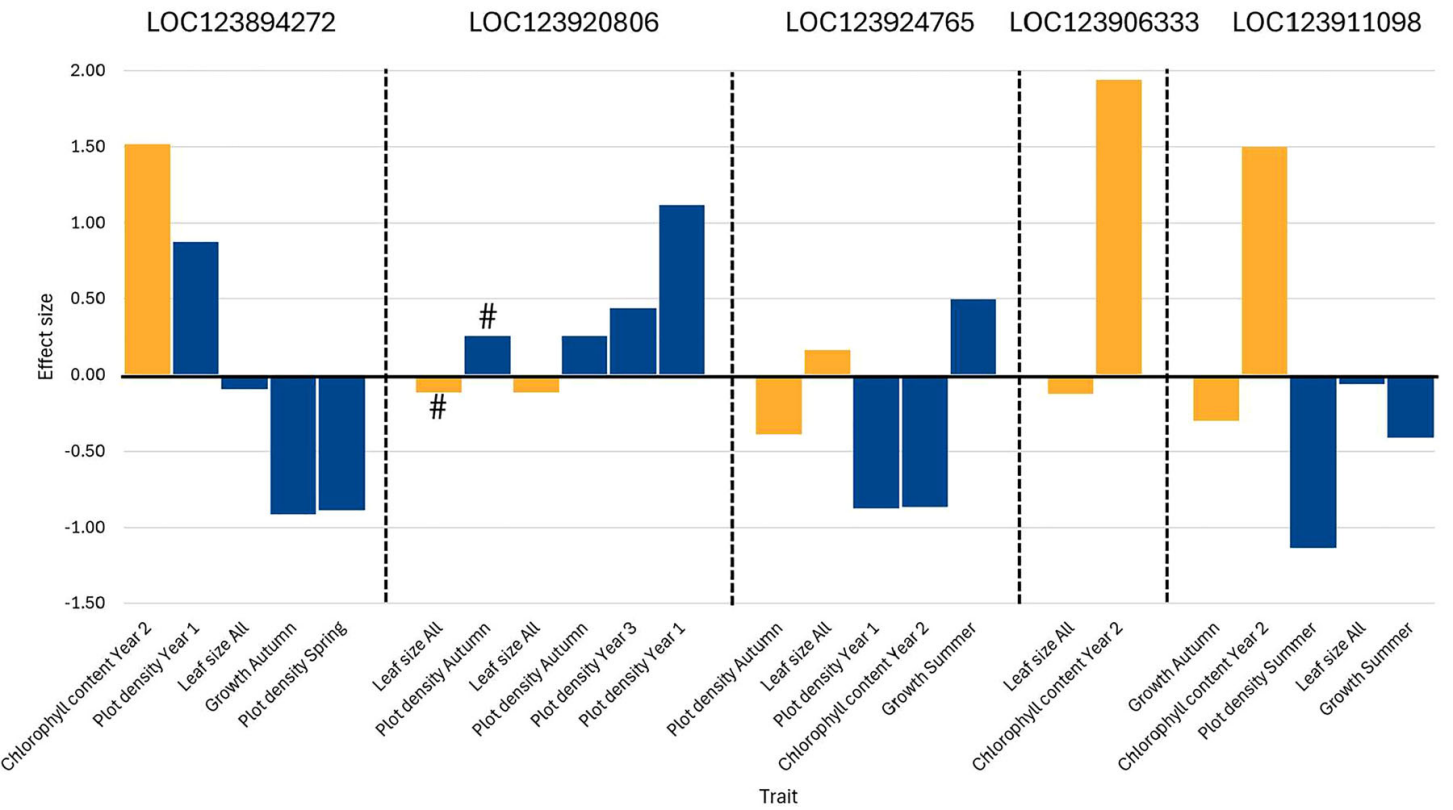
Five exemplar genes of interest in linkage disequilibrium with single nucleotide polymorphisms (SNPs) associated with bioclimatic variables assessed for relationship of allele status and trait value. Direction of bar indicates whether the alternative allele had a negative or positive influence based on SNP allele configuration. Color indicates influence in top five performing half‐sib families (blue) and bottom five performing half‐sib families (yellow) individuals. Locus (LOC) numbers refer to the gene identifier based on annotation gene as described by Bikhart et al. (2022). The hash symbol (#) indicates different SNPs identified for same gene.

The influence on trait performance of the alternate allele was determined for all SNPs, then information for the 77 bioclimatic SNPs was extracted. The 77 SNPs identified as being significantly associated with bioclimatic variable were scanned for associated genes within the estimated LD. For LD calculations, after filtering, a total of 1179 and 1141 SNPs were used to genotype the 40 novel germplasm ecotypes (Gen 0) and 100 half‐sib families (Gen 2), respectively. The mean LD was moderate for both Gen 0 (mean *r* ^2^ = 0.04) and Gen 2 (mean *r* ^2^ = 0.02). LD decay analysis showed a rapid decline in *r* ^2^ with increasing physical distance between SNPs, reaching *r* ^2^ < 0.2 threshold at approximately 5000 bp for Gen 0 and 500 bp for Gen 2. Based on this threshold, five genes were identified as being within LD of the 77 bioclimatic‐associated SNPs (Table S5). These five genes and their influence on trait performance by association with allele configuration are shown in Figure 6. SNPs were located within three of these genes of interest (Table S5). One of these genes, LOC123894272, was predicted to encode a BSD domain‐containing protein implicated in transcriptional regulation, potentially influencing plant development and stress responses. In the top‐performing individuals, the presence of the alternate allele for the SNP associated with this gene was associated with increased plot density in Year 1 but reduced leaf size (Figure 6). The second gene (LOC123920806) was predicted to encode cytoplasmic transmembrane proteins, suggesting a role in membrane transport or signaling. In bottom‐performing individuals, the alternate SNP allele was linked to reduced leaf size, whereas in top performers, it corresponded to increased plot density scores. The third gene was LOC123924765, which was predicted to encode an O‐fucosyltransferase enzyme, which adds fucose residues to proteins and participates in diverse developmental processes including pollen tube growth, plant immunity, and hormone signaling. In bottom‐performing individuals, the alternate allele was associated with larger leaf size but reduced autumn plot density, while in top performers, it was linked to lower Year‐1 plot density and chlorophyll content, but enhanced summer biomass.

The two remaining genes with bioclimatic associated SNPs had SNPs located outside the gene but within LD (Table S5). The first gene (LOC123906333) was predicted to encode a glycoside hydrolase involved in cell wall modification and pathogen defense. Among bottom‐performing individuals, the alternate allele for the SNP associated with this gene corresponded to smaller leaf size but higher chlorophyll content (Figure 6). The second gene (LOC123911098) was predicted to encode a thylakoid luminal protein involved in regulating and stabilizing photosynthetic processes. In bottom‐performing individuals, the alternate SNP allele was associated with reduced autumn biomass and increased chlorophyll content, while in top performers, it was linked to decreases in leaf size, summer biomass, and summer plot density (Figure 6). Finally, four additional genes (LOC123883434, LOC123889838, LOC123900270, and LOC123924713) were identified outside the regions of estimated LD with the associated SNP for the half‐sib families (Table S5).

### 3.4 Genomic prediction identifies candidate traits for selection

**FIGURE 7:**
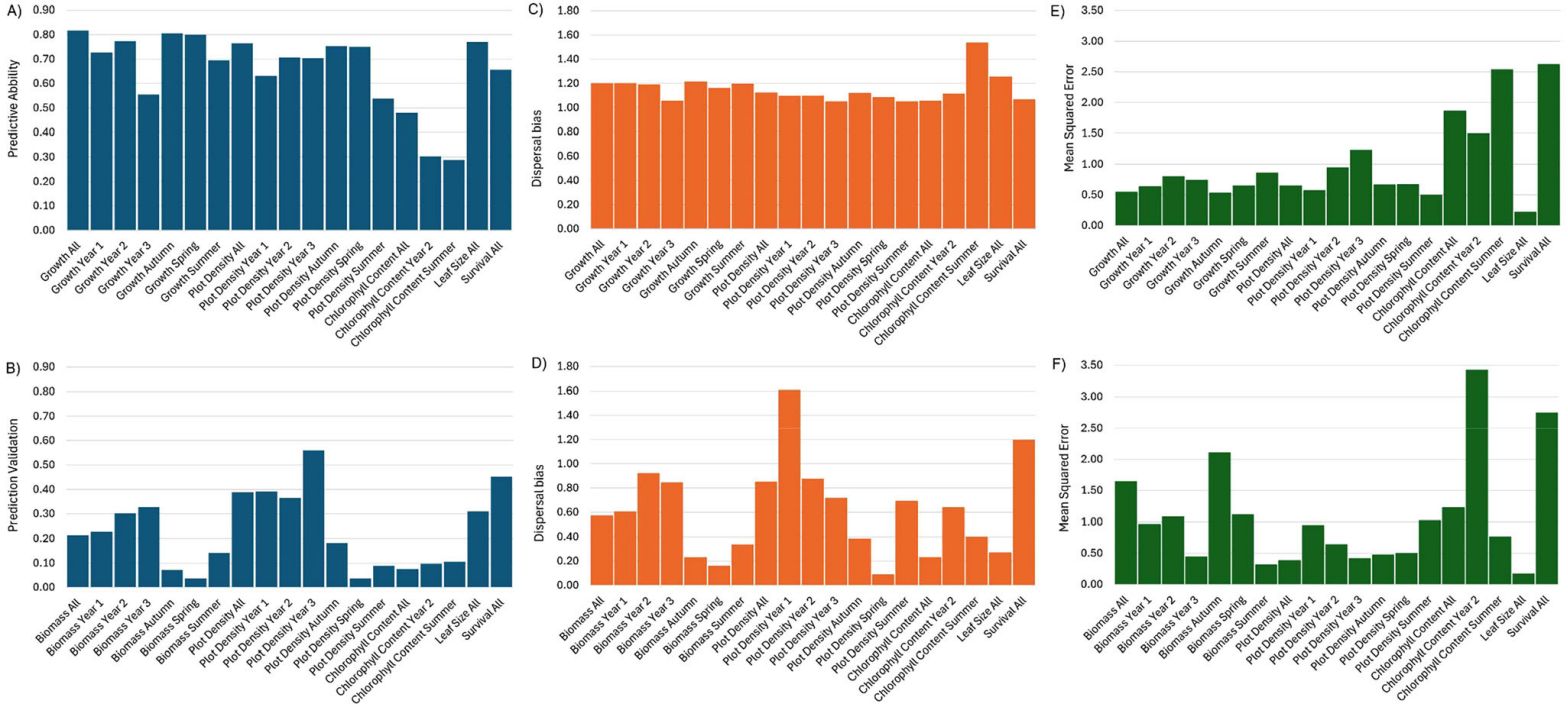
(A) Predictive ability of five traits across years and seasons (19 observations) for the training population (generation zero [Gen 0]) and (B) prediction validation of these same trait observations for generation two (Gen 2) populations. (C) Dispersion bias estimates of the 19 trait observations for the training population (Gen 0) and (D) for the Gen 2 populations. (E) Mean square error estimates of 19 traits for the training population (Gen 0) and (F) for the Gen 2 populations.

Both biomass and plot density traits for Gen 0, excluding biomass in Year 3 and plot density in summer, were found to have high predictive ability in this training set (Figure 7). Leaf size and survival were also found to be suitable for inclusion in a predictive model. Leaf size performed well, as it had the lowest MSE and dispersion bias, which suggested it could provide stable predictions. Chlorophyll content traits performed poorly, possibly due to noisy data as leaf color can vary within and among individual plants. Overall, for all traits dispersion bias values approximated one, suggesting predictions were neither over‐ nor under‐estimated. When across‐generation prediction using the Gen 0 training set was applied to the Gen 2 half‐sib families, an assessment of trait prediction accuracy was made as the GEBVs of the half‐sib families in the field were compared with their realized trait value. The prediction validation, defined as the realized phenotype of the Gen 2 half‐sib families correlated with the GEBVs predicted with the Gen 0 training population (Figure 1), was lower compared to the Gen 0 predictive ability (Figure 7B), although the prediction validation of some traits such as survival (0.45) and some plot density traits (0.4–0.56) were higher than plot density by season and chlorophyll Gen 0 predictive abilities. Overall, traits measured across multiple years had higher prediction validations, whereas seasonal measurements performed worst.

## 4 DISCUSSION

### 4.1 Agronomic and plant performance among generations

Identifying populations with biomass production either at or above both contributing cultivars showed the benefit of the novel genetics from diverse red clover ecotypes that have been introgressed either providing new adaptive traits or new allele combinations that improve such complex traits. For the few C‐derived populations that performed agronomically better than their maternal parent (Colenso), the addition of novel genetics may have boosted performance through heterosis. Additionally, across both R‐ and C‐derived families, none of the Gen 2 populations performed significantly worse than the Colenso. Previously, Heslop et al. (2025) evaluated C‐ and R‐derived Gen 1 balanced bulks, alongside the two cultivars and the 40 ecotypes from which these Gen 1 populations were derived, in multi‐location, multi‐year field trials. These trials showed that several of the diverse ecotypes were well adapted to the local environment and management systems, resulting in biomass production comparable to the cultivars (Heslop et al., 2025; Figure S4). Notably, ecotypes from Cluster 2, which contributed substantially to the genetic composition of the Gen 1 populations, were particularly well adapted to the environmental conditions at the field trial sites (Heslop et al., 2025). This strong performance of the subsequent generation was an important observation as a documented risk of introducing novel germplasm is linkage drag, the inheritance of unwanted alleles that reduce plant performance or maladaptation (Tanksley & Nelson, 1996). Therefore, we can conclude the addition of the 40 novel germplasm genetics did not significantly decrease the agronomic performance of the Gen 2 populations.

Within this study, clear phenotypic differences were observed among top‐ and bottom‐performing Gen 2 families derived from both C‐ and R‐derived Gen 1 populations. However, no significant differences in genetic contributions from Gen 0 ecotypes to these Gen 1 populations were detected. This suggests that different combinations of novel alleles with the locally adapted material to form Gen 1 and subsequent recombination could have underpinned the differential Gen 2 population performance.

### 4.2 Molecular marker genealogy and paternity assessment

The use of predictive models using molecular markers facilitated estimation of the genetic backgrounds of multiple generations, which allowed heritability of traits and, for a select few, the paternity to be tracked. For breeding programs, the ability to track the proportion of genetic backgrounds is important, especially in the introgression of novel germplasm material where geographically and morphologically diverse genetic clusters have been identified. The tracking of lineage can allow weighting to be attributed to clusters depending on the phenotypic expression or performance of those lines for selection into breeding pools. Galeano et al. (2021) identified the benefit of using a GBS‐based parentage analysis in white spruce (*Picea glauca*) by identifying unequal parental contributions and the detection of pollen contamination within the populations. Ensuring quality control of pollen contributions in crosses is crucial, particularly in open‐pollinated crosses, such as in our study, where contamination from stray pollen can occur (Riday, 2011). Through the implementation of a GBS‐based parentage analysis, a breeder can be confident of the genetics within breeding programs.

Mating proficiency is the ability of an individual to contribute genetically to the next generation through successful pollination and seed production. With the introgression of novel germplasm populations into locally adapted populations (maternal individuals), identifying successful or proficient paternal individuals gives several key benefits to breeding programs. It enhances parental selection by identifying effective pollen donors, increasing genetic gain through the selection of individuals with desirable traits, excluding those with undesirable traits, and providing a clearer understanding of gene flow. Riday (2011) showed in red clover that SSR markers empowered selection strategies by being able to use both maternal and paternal breeding value estimates for selection. Using SSR markers to identify both parents, more than double the selection gain was found compared to solely using maternity information (Riday, 2011). The use of GBS for parental assignment was vindicated by Whalen et al. (2019), who found it to be a useful tool whether employing both high‐ or low‐coverage GBS. It was found that knowing one parent and having a large number of markers was advantageous to reduce false positives, where individuals that are not actually parents are identified as such (Whalen et al., 2019). Although resource intense, the findings from this study highlighted the added value of adopting predictive models for estimating paternity for forage breeding programs to improve precision and better introgression of novel germplasm genetics.

### 4.3 Candidate SNPs and phenotypic trait interactions identified

The identification of 37 SNPs that were significantly associated with phenotypic expression and bioclimatic variables provides a route to identifying the associated candidate genes and chromosomal location that could influence plant performance and adaptation (Bragg et al., 2015). A conventional GWAS was not undertaken due to limited effective sample size, strong family structure, and prior candidate SNP selection; instead, phenotypes were stratified into performance classes to enable robust estimation of allele effect size and direction. Both isothermality (Bio3), which reflects temperature stability throughout the year, and the mean temperature of the driest quarter (Bio8) were associated with the highest number of SNPs. Both variables are known to strongly influence plant survival (Heslop et al., 2025; Osterman et al., 2021). In the context of adapting to new environments, reduced temperature variability and similar temperatures between place of origin during periods of water deficit may reduce stress and enhance the plant's adaptive capacity. Most of these SNPs were associated with candidate genes that encode proteins that could be implicated in essential cellular processes such as photosynthesis, transcription, metabolic regulation, and membrane signaling (Table S6). However, further functional characterization and phenotypic evaluation is required to determine their specific contribution to plant performance and adaption (Heslop et al., 2025). Genes LOC123924765 (an O‐fucosyl transferase) and LOC123911098 (a Thylakoid lumenal protein) have likely roles in metabolic regulation, cell wall structure or growth signaling pathways, or photosynthetic efficiency, respectively, and in this study their associations with phenotypic traits indicate a possible connection to biomass accumulation and leaf structure. While genes LOC123894272 (a BSD domain transcription factor) and LOC123920806 (a transmembrane protein) have putative roles in transcription control and membrane signaling, respectively, their associations in this study with phenotypic traits indicate a possible connection to plant architecture and resource allocation. Further functional characterization and phenotypic evaluation is required to determine their specific contribution to plant performance and adaption (Heslop et al., 2025). However, as these adaptive markers have been identified through landscape genomics redundancy analysis in one generation (Gen 0) (Heslop et al., 2025) and now identified two generations later (Gen 2), it further highlights that these genes could be a potential SNP marker linked to adaptation or trait expression. The associations between SNP‐linked genes, bioclimatic variables, and phenotypic expression highlight the importance of further investigating these genomic regions to understand their roles and potential utility in trait improvement through selection.

### 4.4 Genomic prediction potential

The use of pooled rather than individual genotypes in some generations may influence genomic predictive abilities if predictions are interpreted at the individual level. However, in this study, genomic predictions were explicitly made and interpreted at the population level, consistent with the breeding system of red clover. Previous work in outcrossing forage species has demonstrated that pool GBS‐derived allele frequencies can provide reliable population level genomic information (O. G. Ehoche et al., 2025; Faville et al., 2020). When establishing a genomic prediction model, two important factors shaping the model are the number of molecular markers used and the size of the training population. In this present study, 3275 SNP markers were used, while this is smaller than other studies in white clover (110,000 SNPs) (G. O. Ehoche et al., 2021; O. G. Ehoche et al., 2025) and alfalfa (7000–11,000 SNPs) (Annicchiarico et al., 2015) it has been shown to be sufficient by Skøt et al. (2024), who showed having a larger number of SNPs is preferred but also reported that the predictive ability remains stable until marker numbers are below 1000. The size of both the training and validation sets are important considerations in genomic prediction studies.

The smaller sized training population of 92 individuals used in this study would ideally be increased to capture more variation among the traits; however, this was partially offset by selecting such a diverse range of populations. As the size of our validation populations was smaller (50 individuals each) to minimize the population size effect, both C‐derived and R‐derived populations were pooled to reach the minimum of 100 individuals identified by O. G. Ehoche et al. (2025), which showed that the predictive abilities were similar for 100 individuals when reduced from their full set of 200 families, although variability increased as the training populations reduced.

Werner et al. (2020) demonstrated that stable prediction abilities across generations are often driven by persistent population structure and genetic relatedness rather than purely by direct capture of the genetic effects underlying the trait. In structured breeding populations, prediction accuracy can therefore be maintained when individuals in the validation population remain genetically related to those in the training population, allowing models to capture stable family or population level genetic effects transmitted across generations. Ideally, larger training and validation sets are preferred, as additional information reduces variability in marker effect estimation; however, at a minimum, 100 individuals appear sufficient in multi‐site, multi‐year trials (O. G. Ehoche et al., 2025; Resende et al., 2012).

This study used a training population comprised only of novel germplasm to predict the performance of breeding lines two generations later. This is of significance, as having a close genetic relationship between training and validation population sets is important for accuracy. The training population had high predictive abilities for many complex traits, such as biomass and plot survival with several at approximately 0.8. This is higher than predictive models for populations derived from more elite, hence less “raw” material. For example, O. G. Ehoche et al. (2025) working with half‐sib white clover families in a multi‐year, multi‐site field trial calculated predictive abilities of 0.2 and 0.3 for dry matter yield and biomass, respectively. Such high predictive abilities in the red clover novel germplasm may be due to the wide phenotypic diversity identified, and similar results have been found in pea (*Pisum sativum* L.) and maize (*Zea mays* L.) germplasm (Bari et al., 2021; Heslop et al., 2025; Rogers et al., 2022).

Developing a red clover prediction model trained at Gen 0 and applying it to material two generations later (Gen 2) was an opportunity to assess model longevity. Furthermore, as the Gen 2 families were also tested in the field, the correlation between GEBVs and validation in the field provided prediction validation data. While there were two generations between the training set and the validation set, the correlations of the prediction model and validation results were lower than the predictive abilities but were respectable for some traits such as survival (0.45) and some plot density traits (0.4–0.56). These reduced correlations are likely attributable to genotype by environment interactions and to recombination between marker alleles and causative genomic regions across generations, which can erode prediction accuracy. Additionally, differing environmental conditions between data collection for training and validation populations, that is, drier summers during model training and wetter summers during validation, can contribute to lower prediction accuracy; nevertheless, correlations for survival and plot density remained respectable. This suggests a route to utilizing genomic selection for improvement when incorporating exotic germplasm into breeding lines.

In this study, the heritability levels were found to be higher for plot density and biomass traits, and it was also apparent that the training population had greater heritability values compared to the validation population. This could be due to the nature of the two populations with the training population being raw germplasm with high genetic diversity creating higher values, while the training population had narrower genetic diversity due to being breeding lines. This suggests that the genetic architecture of traits in the training population is more variable and potentially more informative for selection, while the reduced diversity in the validation population may limit the expression of genetic variance due to prior selection. Previous studies in field peas (*Pisum sativum* L.), which used training populations comprised of novel germplasm found their prediction models were challenged due to the narrowing of genetics in the breeding lines compared to the genetically diverse germplasm material (Bari et al., 2021; Crosta et al., 2023).

The significance of comparative evaluation environments for training and validation populations was also highlighted in field peas where differences in sowing times and exposure to drought stress over trial durations impacted the performance of the prediction model (Bari et al., 2021; Crosta et al., 2023). Similar trends occurred in our populations where lower heritability and predictive ability were found for traits more prone to variation caused by higher genotype‐by‐environment interactions, such as chlorophyll content and seasonal biomass and plot density. This highlighted the importance of collecting good‐quality phenotypic data from multiple locations, seasons, and years for traits more prone to the effects of genotype‐by‐environment interactions. The benefit of combining data across locations for genomic prediction was also shown to improve predictive ability, and this has also been found in red clover (Skøt et al., 2024), perennial ryegrass (*Lolium perenne* L.; Faville et al., 2018.), alfalfa (*Medicago sativa* L.; Annicchiarico et al., 2015), white clover (*Trifolium repens* L.; O. G. Ehoche et al., 2025), and rice (*Oryza sativa* L.; Spindel et al., 2015).

A trend observed as the trial matured was that the predictive ability for biomass traits increased, and similar trends have been reported in perennial ryegrass (Grinberg et al., 2016) and white clover (O. G. Ehoche et al., 2025). Plot density traits, however, remained similar across years, potentially reflecting higher additive genetic variation due to more confounding factors across the years with higher predictive ability. For white clover it was suggested possible confounding factors could either be the establishment period of the trial, which often involves transplanting, which can disturb plant growth, or the transition from a taproot to the stoloniferous root system (O. G. Ehoche et al., 2025). This could be similar for red clover; however, instead of transitioning from a taproot system, red clover builds its taproot system over time prioritizing root growth over shoot growth. Given the lifespan of red clover is 3–5 years, once the taproot system is optimized in the 18 months shoot growth can increase or better reflect its true phenotypic and agronomic potential. As suggested for white clover, a minimum of 3 years should be used to collect phenotypic data for red clover as this should be a true reflection on plant trends, agronomic potential, and persistence (O. G. Ehoche et al., 2025).

Previously, only one genomic prediction study had been carried out for red clover by Skøt et al. (2024), which compared different predictive models for dry matter yield, crude protein, and date of flowering. While there are no published genomic prediction values for visual biomass, it is highly correlated to biomass yield or dry matter yield (Riday, 2009). In this case, we can compare our predicted accuracies to that of other trials that just measure dry matter yield. Skøt et al. (2024) found a wide range (0.40–0.87) of predictive ability for dry matter yield, which fluctuated across years and seasons. While in other legumes, 0.30 was found for both biomass yield and dry matter yield in white clover, and 0.32–0.35 and 0.13 in two separate studies for biomass yield in alfalfa (Annicchiarico et al., 2015; G. O. Ehoche et al., 2021, 2025; Jia et al., 2018). Further, for perennial ryegrass, predictive ability across multiple studies was found to range between 0.013 and 0.59 (Faville et al., 2018; Grinberg et al., 2016; Pembleton et al., 2018). Interestingly, although the predictive values were at the lower level, as found in our study, selection based on GEBVs produced at this level is projected to produce greater genetic gain compared to straight phenotypic selection in both white clover and perennial ryegrass (G. O. Ehoche et al., 2021; Faville et al., 2020).

### 4.5 Conclusion and next steps

This study assessed the introgression of novel genetics via the crossing of germplasm material into locally adapted elite populations. Genetic variation and heritability of key agronomic and physiological traits were assessed in both the germplasm material and their offspring, using genetic analyses based on half‐sib family performance across multiple locations and years, using field trials. The benefit of the introgression of novel genetics was apparent through the higher agronomic performance of some Gen 2 populations compared to their maternal cultivars. The identification of adaptive markers associated to phenotypic expression highlighted previously through landscape genomics was of significance, with further investigation required to establish the suitability of these SNPs as molecular markers. This research demonstrates the potential of harnessing the genetic diversity within red clover germplasm through genomic prediction, providing an efficient pathway to identify valuable material for breeding programs and deliver cultivars that combine agronomic performance with future climate resilience.

## AUTHOR CONTRIBUTIONS

**A. D. Heslop**: Conceptualization; data curation; formal analysis; funding acquisition; investigation; methodology; visualization; writing—original draft; writing—review and editing. **S. K. Arojju**: Conceptualization; investigation; methodology; supervision; visualization; writing—review and editing. **R. W. Hofmann**: Conceptualization; investigation; methodology; supervision; visualization; writing—review and editing. **J. L. Ford**: Conceptualization; investigation; methodology; supervision; visualization; writing—review and editing. **C. A. Hefer**: Investigation; methodology; writing—review and editing. **M. Z. Z. Jahufer**: Conceptualization; investigation; methodology; supervision; visualization; writing—review and editing. **A. C. Larking**: Investigation; methodology; writing—review and editing. **W. Hong**: Investigation; methodology; writing—review and editing. **T. P. Bilton**: Investigation; methodology; writing—review and editing. **R. Ashby**: Investigation; methodology; writing—review and editing. **J. O'Connor**: Investigation; methodology; writing—review and editing. **A. G. Griffiths**: Conceptualization; investigation; methodology; supervision; writing—review and editing.

## CONFLICT OF INTEREST STATEMENT

Authors J. L. Ford and S. K. Arojju are employed by PGG Wrightson Seeds Ltd and Radiata Pine Breeding Company, respectively. The remaining authors declare no conflicts of interest.

## Supporting information



Supporting Information

## Data Availability

The raw data supporting the conclusions of this article will be made available by the authors, without undue reservation. The datasets presented in this study can be found at https://github.com/Angu5H/Harnessing‐exotic‐germplasm.
