# Supplementary material for: Harnessing exotic germplasm for red clover improvement: Tracking genomic introgression and deploying genomic selection
Source: Plant Genome. 2026 Aug 18;19(3):e70287. doi: 10.1002/tpg2.70287 (PMC13492578; doi:10.1002/tpg2.70287)
Supplement: Supplementary file 1 [file TPG2-19-e70287-s001.docx]

# **Supplementary data**

**Supplementary Table 1** 40 accessions and their source country identifiers and their genetic clusters identified in Heslop et al., 2025. Arm = Armenia; Azb = Azerbaijan; Geo = Georgia; Gre = Greece; Por = Portugal; Rus = Russia; Spa = Spain; Taj = Tajikistan

| Cluster 1 | Cluster 2 | Cluster 3 | Cluster 4 | Cluster 5 | Cluster 6 | Cluster 7 |
| --- | --- | --- | --- | --- | --- | --- |
| Por51 | Arm4 | Arm7 | Taj69 | Gre32 | Rus22 | Por46 |
| Por53 | Arm5 | Arm8 | Taj71 | Gre33 | Geo24 | Por47 |
| Spa62 | Azb13 | Arm9 | Taj73 | Gre34 |  | Por52 |
| Spa65 | Rus20 | Arm11 | Taj74 | Gre36 |  | Spa61 |
| Spa66 | Rus23 | Azb14 | Taj76 | Gre37 |  |  |
| Spa67 | Por49 | Azb15 | Taj77 | Gre38 |  |  |
|  | Spa64 | Azb16 |  |  |  |  |
|  |  | Azb26 |  |  |  |  |
|  |  | Azb27 |  |  |  |  |

**Supplementary Figure 1** Biomass BLUPs across three years at both locations for A) Colenso-derived populations relative to red clover cultivars ‘Colenso’ and ‘Relish’. Yellow and Red dash lines show upper and lower quartile of parent cultivar. B) Relish-derived populations relative to red clover cultivars ‘Colenso’ and ‘Relish’. Yellow and Red dash lines show upper and lower quartile of parent cultivar. Purple dash line shows upper quartile of Colenso parent in comparison to R-derived populations. Error bars represent ± least significant differences (LSDs).

**Supplementary Table 2** DAPC proportion estimates of the C-derived Gen 2 individuals

**Supplementary Table 3** DAPC proportion estimates of the R-derived Gen 2 individuals

**Supplementary Figure 2** The average proportion inherited from Gen 1 ecotypes found in top five and bottom five C-derived Gen 2 and R-derived Gen 2 individuals for biomass, density and survival across years and seasons, grouped on an optimum of seven clusters identified through DAPC analysis.

**Supplementary Figure 3** Mating proficiency estimates for the half-sib families **A)** Colenso 38 (C38), **B)** Relish 1 (R1) and **C)** Relish 30 (R30) that had genotype-by-sequence material collected and processed individually.

**Supplementary Table 4** SNPs and associated genes that have significant association to the phenotypic expression of particular traits

| **SNP** | **Trait** | **ID** | **Effect size** | **Direction** | **Gene** |
| --- | --- | --- | --- | --- | --- |
| NC_060059.1_27965571 | Chlorophyll Content Summer | Group_Bottom | -0.30 | Decrease caused by Alternate Allele | LOC123924713 |
| NC_060059.1_27965571 | Plot Density Year 1 | Group_Top | -0.50 | Decrease caused by Alternate Allele | LOC123924713 |
| NC_060059.1_27965571 | Plot Density Year 3 | Group_Top | -0.31 | Decrease caused by Alternate Allele | LOC123924713 |
| NC_060059.1_27965571 | Biomass Autumn | Group_Top | 0.56 | Increase caused by Alternate Allele | LOC123924713 |
| NC_060059.1_27965571 | Plot Density Summer | Group_Top | 0.85 | Increase caused by Alternate Allele | LOC123924713 |
| NC_060059.1_27965571 | Plot Density Autumn | Group_Top | 0.37 | Increase caused by Alternate Allele | LOC123924713 |
| NC_060059.1_27965571 | Plot Density Spring | Group_Top | 0.53 | Increase caused by Alternate Allele | LOC123924713 |
| NC_060059.1_27965571 | Leaf Size All | Group_Top | 0.06 | Increase caused by Alternate Allele | LOC123924713 |
| NC_060059.1_27965571 | Biomass Summer | Group_Top | 0.27 | Increase caused by Alternate Allele | LOC123924713 |
| NC_060059.1_35463981 | Leaf Size All | Group_Bottom | -0.11 | Decrease caused by Alternate Allele | LOC123920806 |
| NC_060059.1_35463981 | Plot Density Autumn | Group_Bottom | 0.26 | Increase caused by Alternate Allele | LOC123920806 |
| NC_060059.1_35463985 | Leaf Size All | Group_Bottom | -0.11 | Decrease caused by Alternate Allele | LOC123920806 |
| NC_060059.1_35463985 | Plot Density Year 3 | Group_Bottom | 0.44 | Increase caused by Alternate Allele | LOC123920806 |
| NC_060059.1_35463985 | Plot Density Autumn | Group_Bottom | 0.26 | Increase caused by Alternate Allele | LOC123920806 |
| NC_060059.1_35463985 | Plot Density Year 1 | Group_Bottom | 1.12 | Increase caused by Alternate Allele | LOC123920806 |
| NC_060059.1_8414746 | Plot Density All | Group_Bottom | 0.84 | Increase caused by Alternate Allele | LOC123918922 |
| NC_060059.1_8414746 | Plot Density Year 2 | Group_Bottom | 1.24 | Increase caused by Alternate Allele | LOC123918922 |
| NC_060059.1_8414746 | Biomass Year 2 | Group_Bottom | 1.15 | Increase caused by Alternate Allele | LOC123918922 |
| NC_060059.1_9786448 | Plot Density Year 1 | Group_Top | 0.93 | Increase caused by Alternate Allele | LOC123920368 |
| NC_060060.1_14151536 | Biomass Autumn | Group_Bottom | -0.30 | Decrease caused by Alternate Allele | LOC123907412 |
| NC_060060.1_18091886 | Biomass Autumn | Group_Bottom | 0.20 | Increase caused by Alternate Allele | LOC123903762 |
| NC_060060.1_36381293 | Biomass Spring | Group_Bottom | 0.56 | Increase caused by Alternate Allele | LOC123905879 |
| NC_060060.1_39413353 | Plot Density Year 1 | Group_Top | -0.57 | Decrease caused by Alternate Allele | LOC123907287 |
| NC_060060.1_39413381 | Chlorophyll Content All | Group_Top | -1.30 | Decrease caused by Alternate Allele | LOC123907287 |
| NC_060060.1_40809068 | Biomass Autumn | Group_Bottom | -0.30 | Decrease caused by Alternate Allele | LOC123911098 |
| NC_060060.1_40809068 | Chlorophyll Content Year 2 | Group_Bottom | 1.50 | Increase caused by Alternate Allele | LOC123911098 |
| NC_060060.1_40809068 | Plot Density Summer | Group_Top | -1.14 | Decrease caused by Alternate Allele | LOC123911098 |
| NC_060060.1_40809068 | Leaf Size All | Group_Top | -0.06 | Decrease caused by Alternate Allele | LOC123911098 |
| NC_060060.1_40809068 | Biomass Summer | Group_Top | -0.41 | Decrease caused by Alternate Allele | LOC123911098 |
| NC_060060.1_65980169 | Leaf Size All | Group_Bottom | -0.12 | Decrease caused by Alternate Allele | LOC123906333 |
| NC_060060.1_65980169 | Chlorophyll Content Year 2 | Group_Bottom | 1.94 | Increase caused by Alternate Allele | LOC123906333 |
| NC_060062.1_15837500 | Biomass Spring | Group_Top | -1.29 | Decrease caused by Alternate Allele | LOC123924698 |
| NC_060062.1_15837500 | Biomass All | Group_Top | -0.29 | Decrease caused by Alternate Allele | LOC123924698 |
| NC_060062.1_15837500 | Plot Density Spring | Group_Top | -0.58 | Decrease caused by Alternate Allele | LOC123924698 |
| NC_060062.1_26187694 | Plot Density Autumn | Group_Bottom | -0.39 | Decrease caused by Alternate Allele | LOC123924765 |
| NC_060062.1_26187694 | Leaf Size All | Group_Bottom | 0.17 | Increase caused by Alternate Allele | LOC123924765 |
| NC_060062.1_26187694 | Plot Density Year 1 | Group_Top | -0.87 | Decrease caused by Alternate Allele | LOC123924765 |
| NC_060062.1_26187694 | Chlorophyll Content Year 2 | Group_Top | -0.86 | Decrease caused by Alternate Allele | LOC123924765 |
| NC_060062.1_26187694 | Biomass Summer | Group_Top | 0.50 | Increase caused by Alternate Allele | LOC123924765 |
| NC_060062.1_4099588 | Plot Density All | Group_Top | -0.18 | Decrease caused by Alternate Allele | LOC123881749 |
| NC_060062.1_50300966 | Chlorophyll Content Summer | Group_Top | -0.43 | Decrease caused by Alternate Allele | LOC123882380 |
| NC_060062.1_50300975 | Chlorophyll Content Summer | Group_Top | -0.43 | Decrease caused by Alternate Allele | LOC123882380 |
| NC_060062.1_52789750 | Plot Density All | Group_Bottom | -0.74 | Decrease caused by Alternate Allele | LOC123924416 |
| NC_060062.1_52789750 | Biomass All | Group_Bottom | -0.28 | Decrease caused by Alternate Allele | LOC123924416 |
| NC_060062.1_52789750 | Chlorophyll Content Year 2 | Group_Bottom | -2.43 | Decrease caused by Alternate Allele | LOC123924416 |
| NC_060062.1_52789750 | Plot Density Year 1 | Group_Top | -0.87 | Decrease caused by Alternate Allele | LOC123924416 |
| NC_060063.1_16749858 | Biomass Autumn | Group_Bottom | -0.18 | Decrease caused by Alternate Allele | LOC123885270 |
| NC_060063.1_16749858 | Biomass Summer | Group_Bottom | 0.27 | Increase caused by Alternate Allele | LOC123885270 |
| NC_060063.1_16749861 | Biomass Autumn | Group_Bottom | -0.18 | Decrease caused by Alternate Allele | LOC123885270 |
| NC_060063.1_41477381 | Leaf Size All | Group_Bottom | -0.16 | Decrease caused by Alternate Allele | LOC123886679 |
| NC_060063.1_41478392 | Plot Density Year 2 | Group_Bottom | 0.99 | Increase caused by Alternate Allele | LOC123886679 |
| NC_060063.1_53390230 | Plot Density All | Group_Bottom | -0.55 | Decrease caused by Alternate Allele | LOC123883037 |
| NC_060063.1_53390230 | Biomass All | Group_Bottom | -0.28 | Decrease caused by Alternate Allele | LOC123883037 |
| NC_060063.1_53390230 | Plot Density Year 2 | Group_Bottom | -1.10 | Decrease caused by Alternate Allele | LOC123883037 |
| NC_060063.1_53390230 | Plot Density Year 1 | Group_Bottom | -1.11 | Decrease caused by Alternate Allele | LOC123883037 |
| NC_060063.1_53390230 | Biomass Year 2 | Group_Bottom | -1.05 | Decrease caused by Alternate Allele | LOC123883037 |
| NC_060063.1_53804064 | Biomass Year 3 | Group_Bottom | -0.44 | Decrease caused by Alternate Allele | LOC123884770 |
| NC_060063.1_53804064 | Plot Density Year 3 | Group_Top | -0.55 | Decrease caused by Alternate Allele | LOC123884770 |
| NC_060063.1_53804085 | Plot Density Year 3 | Group_Top | -0.55 | Decrease caused by Alternate Allele | LOC123884770 |
| NC_060063.1_53804085 | Plot Density Summer | Group_Top | 1.35 | Increase caused by Alternate Allele | LOC123884770 |
| NC_060063.1_55473352 | Chlorophyll Content Summer | Group_Bottom | -0.33 | Decrease caused by Alternate Allele | LOC123883338 |
| NC_060063.1_57674718 | Plot Density Summer | Group_Top | 1.41 | Increase caused by Alternate Allele | LOC123883434 |
| NC_060063.1_57674734 | Plot Density Year 2 | Group_Bottom | -0.99 | Decrease caused by Alternate Allele | LOC123883434 |
| NC_060063.1_57674804 | Plot Density Year 2 | Group_Bottom | -0.58 | Decrease caused by Alternate Allele | LOC123883434 |
| NC_060063.1_57674804 | Chlorophyll Content Year 2 | Group_Bottom | -1.96 | Decrease caused by Alternate Allele | LOC123883434 |
| NC_060063.1_57674804 | Plot Density Year 1 | Group_Top | -0.58 | Decrease caused by Alternate Allele | LOC123883434 |
| NC_060063.1_57674804 | Biomass Autumn | Group_Top | 0.71 | Increase caused by Alternate Allele | LOC123883434 |
| NC_060063.1_57674804 | Biomass Spring | Group_Top | 1.72 | Increase caused by Alternate Allele | LOC123883434 |
| NC_060063.1_57692453 | Biomass Spring | Group_Top | 1.74 | Increase caused by Alternate Allele | LOC123883439 |
| NC_060063.1_57692453 | Plot Density Autumn | Group_Top | 0.45 | Increase caused by Alternate Allele | LOC123883439 |
| NC_060064.1_17529215 | Chlorophyll Content Year 2 | Group_Bottom | -1.41 | Decrease caused by Alternate Allele | LOC123889838 |
| NC_060064.1_17529215 | Plot Density Year 1 | Group_Top | -0.61 | Decrease caused by Alternate Allele | LOC123889838 |
| NC_060064.1_17529215 | Biomass Spring | Group_Top | 1.73 | Increase caused by Alternate Allele | LOC123889838 |
| NC_060064.1_17529215 | Plot Density Spring | Group_Top | 0.89 | Increase caused by Alternate Allele | LOC123889838 |
| NC_060064.1_17529215 | Biomass Year 2 | Group_Top | 0.30 | Increase caused by Alternate Allele | LOC123889838 |
| NC_060064.1_8396421 | Plot Density Spring | Group_Top | 0.62 | Increase caused by Alternate Allele | LOC123891580 |
| NC_060065.1_10128232 | Plot Density Summer | Group_Bottom | -1.20 | Decrease caused by Alternate Allele | LOC123900270 |
| NC_060065.1_10128232 | Biomass Autumn | Group_Bottom | -0.20 | Decrease caused by Alternate Allele | LOC123900270 |
| NC_060065.1_10128232 | Plot Density Year 3 | Group_Bottom | 0.44 | Increase caused by Alternate Allele | LOC123900270 |
| NC_060065.1_10128232 | Biomass All | Group_Bottom | 0.29 | Increase caused by Alternate Allele | LOC123900270 |
| NC_060065.1_10128232 | Plot Density Year 1 | Group_Bottom | 0.82 | Increase caused by Alternate Allele | LOC123900270 |
| NC_060065.1_10128232 | Chlorophyll Content Year 2 | Group_Top | 0.84 | Increase caused by Alternate Allele | LOC123900270 |
| NC_060065.1_34908787 | Plot Density Summer | Group_Bottom | 1.31 | Increase caused by Alternate Allele | LOC123896811 |
| NC_060065.1_34908787 | Leaf Size All | Group_Bottom | 0.17 | Increase caused by Alternate Allele | LOC123896811 |
| NC_060065.1_7445552 | Chlorophyll Content Year 2 | Group_Bottom | 1.52 | Increase caused by Alternate Allele | LOC123894272 |
| NC_060065.1_7445552 | Biomass Autumn | Group_Top | -0.91 | Decrease caused by Alternate Allele | LOC123894272 |
| NC_060065.1_7445552 | Plot Density Spring | Group_Top | -0.89 | Decrease caused by Alternate Allele | LOC123894272 |
| NC_060065.1_7445552 | Leaf Size All | Group_Top | -0.09 | Decrease caused by Alternate Allele | LOC123894272 |
| NC_060065.1_7445552 | Plot Density Year 1 | Group_Top | 0.87 | Increase caused by Alternate Allele | LOC123894272 |

**Supplementary Table 5** The features, domains and predicted function of the five exemplar genes with significant associations to phenotypic traits.

| **Gene ID** | **Chromosome** | **Domains / Features** | **Localization** | **Predicted Function** |
| --- | --- | --- | --- | --- |
| LOC123924713 | LG1 | Acyl-CoA N-acyltransferases; Acetyltransferase; GNAT; NAT-SF | – | GNAT superfamily acetyltransferase, involved in protein/metabolite acetylation; may regulate chromatin or secondary metabolism. |
| LOC123920806 | LG1 | Transmembrane protein,  Before translational start site | Cytoplasm (near TSS) | Membrane-spanning protein; likely role in transport or signaling (uncharacterized). |
| LOC123924765 | LG4 | O-fucosyltransferase; GDP-fucose protein, In intron with no effect on protein | Cytoplasm | O-fucosyltransferase; catalyzes protein fucosylation, important for cell wall biosynthesis and signaling. |
| LOC123911098 | LG2 | Thylakoid lumenal protein TL20.3; Chloroplastic; Pentapeptide repeat | Chloroplast (thylakoid lumen) | Thylakoid lumenal protein TL20.3, contributes to photosystem II assembly and photosynthetic efficiency. |
| LOC123894272 | LG7 | BSD domain; BTF2-like transcription factor | Likely nuclear | BSD domain transcription factor; probable role in transcriptional regulation, development, or stress response. |

**Supplementary Figure 4** Biomass BLUPs from Heslop et al., 2025, for 40 Gen 0 ecotypes with two parent cultivars Colenso (Col) and Relish (Rel) and two balanced bulk lines of C-derived (C-Gen1) (unpublished data) across three years at two locations. Error bars represent ± least significant differences (LSDs).
